# A Tempered Particle Filter to Enhance the Assimilation of SAR‐Derived Flood Extent Maps Into Flood Forecasting Models

**DOI:** 10.1029/2022WR031940

**Published:** 2022-08-17

**Authors:** Concetta Di Mauro, Renaud Hostache, Patrick Matgen, Ramona Pelich, Marco Chini, Peter Jan van Leeuwen, Nancy Nichols, Günter Blöschl

**Affiliations:** ^1^ Luxembourg Institute of Science and Technology Luxembourg Italy; ^2^ Institut de Recherche pour le Développement UMR Espace‐Dev University of Réunion University of Guyane University of Antilles University of Nouvelle Calédonie UPVD Montpellier France; ^3^ Department of Meteorology University of Reading Reading UK; ^4^ Department of Atmospheric Science Colorado State University Fort Collins CO USA; ^5^ Department of Mathematics and Statistics University of Reading Reading UK; ^6^ Centre for Water Resource Systems Vienna University of Technology Vienna Austria; ^7^ Institute of Hydrology and Water Resources Management Vienna University of Technology Vienna Austria

**Keywords:** data assimilation, flood model, particle filter, tempering, degeneracy, flood extent map

## Abstract

Data assimilation (DA) is a powerful tool to optimally combine uncertain model simulations and observations. Among DA techniques, the particle filter (PF) has gained attention for its capacity to deal with nonlinear systems and for its relaxation of the Gaussian assumption. However, the PF may suffer from degeneracy and sample impoverishment. In this study, we propose an innovative approach, based on a tempered particle filter (TPF), aiming at mitigating PFs issues, thus extending over time the assimilation benefits. Probabilistic flood maps derived from synthetic aperture radar data are assimilated into a flood forecasting model through an iterative process including a particle mutation in order to keep diversity within the ensemble. Results show an improvement of the model forecasts accuracy, with respect to the Open Loop: on average the root mean square error (RMSE) of water levels decrease by 80% at the assimilation time and by 60% 2 days after the assimilation. A comparison with the Sequential Importance Sampling (SIS) is carried out showing that although SIS performances are generally comparable to the TPF ones at the assimilation time, they tend to decrease more quickly. For instance, on average TPF‐based RMSE are 20% lower compared to the SIS‐based ones 2 days after the assimilation. The application of the TPF determines higher critical success index values compared to the SIS. On average the increase in performances lasts for almost 3 days after the assimilation. Our study provides evidence that the application of the variant of the TPF enables more persistent benefits compared to the SIS.

## Key Points

- We assimilate flood extent maps into a flood forecasting system using a tempered particle filter (TPF)
- The TPF mitigates degeneracy and enables long‐lasting forecast improvements
- The TPF outperforms a standard particle filter in terms of accuracy of model outputs

## 1 Introduction

Every year, floods cause important social and economic losses and the trend is increasing. Tellman et al. (2021) show that worldwide the population exposed to floods has increased by 20%–24% from 2000 to 2015, thereby highlighting the need for accurate and timely forecasts of water depth, discharge, flood wave propagation, and flood extent to help reducing or preventing the adverse effects of floods. Flood forecasting models are commonly used to generate short‐to mid‐term predictions. However, the accuracy of such predictions can be affected by multiple factors contributing to the overall model uncertainty. This challenge represents one of the major unsolved scientific problems (Blöschl et al., 2019). The assimilation of independent observations, such as field gauging data or satellite observations, can help reducing these uncertainties (Liu & Gupta, 2007). The last decade has seen a substantial increase in the number of Earth Observation satellites providing a synoptic overview of the flooding situation at increasingly high frequency. Despite possible errors in the interpretation of the synthetic aperture radar (SAR) data (Chen et al., 2018; Grimaldi et al., 2020; Zhao et al., 2021) that should be masked out before any use of these data, frequent observations of flood extent and water depth represent substantial added value, especially over poorly gauged or ungauged catchments. For example, SAR data are relevant for observing inundation extent because of their day‐night and quasi all‐weather capability. As a consequence, several methods enabling an effective assimilation of such observations (e.g., Andreadis & Schumann, 2014; Garcia‐Pintado et al., 2015; Hostache et al., 2018; Revilla‐Romero et al., 2016) for improving the predictive capability of flood models have been introduced and investigated in recent years. The most widely used methods are based on the Kalman Filter and its variants (e.g., Annis et al., 2021; Revilla‐Romero et al., 2016; Wongchuig‐Correa et al., 2020) and they assume that the distributions of observation and model errors are Gaussian, which is not often the case when dealing with real word data (van Leeuwen et al., 2019).

Particle filters (PFs) have gained attention within the research community because of their ability to handle nonlinear and non‐Gaussian systems (van Leeuwen et al., 2019). PFs approximate the prior and the posterior probability distribution functions (PDFs) with an ensemble of model states also called particles. An equal weight is assigned to each particle a priori. Next, as a result of the assimilation, weights are updated to represent the posterior probability given the observations. The principal limitation of PFs is the difficulty to deal with high‐dimensional systems. The weights may vary significantly across particles and in the ultimate case only one particle will have a weight close to unity while the other particles will have negligible weight. As a result, the ensemble may collapse. This well‐known issue in PFs is often referred to as degeneracy. Degeneracy could lead to an erroneous approximation of the posterior distribution (García‐Pintado et al., 2013) and a sub‐optimal use of the assimilation filter. Resampling methods (e.g., Gordon et al., 1993) have been used to prevent the collapse of the ensemble: particles with significant weights are replicated and non‐significant particles are discarded. Even though resampling is powerful in reducing degeneracy, it often comes with a sample impoverishment and a poor representation of the actual uncertainty of the system (Moradkhani et al., 2012). After few iterations, replicated particles will hardly diversify and particles will again collapse into a single or few particles. According to Snyder et al. (2008), the number of particles should grow exponentially with the dimension of the system, otherwise, the PF may suffer from degeneracy. Of course, a higher number of particles implies an increased computational cost which may hamper the use of DA in near real‐time application. As a consequence, it is important to minimize the weight variance so that each particle keeps a significant weight.

Di Mauro et al. (2021) and Hostache et al. (2018) recently developed, following a similar previous work by Giustarini et al. (2011), a data assimilation (DA) framework based on Sequential Importance Sampling (SIS), a variant of PFs that enables an efficient assimilation of SAR data into a hydrodynamic model. In their experiment, the rainfall forcing and the SAR data are assumed to represent the only sources of uncertainty. While Di Mauro et al. (2021) showed that the SIS method provides good results when the assumptions are indeed satisfied, they also highlight the need for a method to mitigate degeneracy and sample impoverishment. The assimilation via an SIS tends to degenerate with only a few particles getting significant weights as a result of the assimilation. A preliminary attempt to mitigate the degeneracy consisted in using a tempering coefficient for the inflation of the posterior probability. The likelihood was raised to the power of a coefficient whose value enables a substantial increase of the likelihood variance. However, using this coefficient to inflate the likelihood only partially solved the degeneracy issue, and sometimes at the cost of a decrease in prediction accuracy.

To mitigate the mentioned PF‐related issues, the following approaches have been introduced in the literature:

1. Using a one‐step proposal density to steer particles in such a way that they obtain similar weights (Doucet et al., 2001; Van Leeuwen, 2009);
2. Moving the particles from the prior to the posterior by applying a smooth iterative transition process using model transitional densities (Beskos et al., 2014).
3. Using particles filters within Monte‐Carlo Markov Chains (Andrieu et al., 2010)

These methodologies are exact in the limit of an infinite ensemble size. Many approximate algorithms exist, and the following list provides relevant examples of applications in hydrological sciences:

1. Localizing PFs, in which observations are only allowed to influence nearby elements of the state vector (Reich, 2013; Van Leeuwen, 2009);
2. Bringing in approximate elements of ensemble Kalman filters into the PF (Frei & Kunsch, 2013; Potthast et al., 2019);
3. Using approximate Markov Chain Monte Carlo (MCMC) steps within the PF proposal step (PF‐MCMC; Moradkhani et al., 2012);
4. Combining the PF with metaheuristic‐algorithms from Computer Science, such as genetic algorithm (GA; Kwok et al., 2005; Park et al., 2010), particle swarm optimization (Li et al., 2005; Wang et al., 2006), and the immune genetic algorithm (Han et al., 2011);
5. Combining the MCMC with GA algorithms and use it within the importance sampling step of the PF‐MCMC, known as Evolutionary Particle Filter with Markov Chain Monte Carlo (EPFM; Abbaszadeh et al., 2018);
6. Using 4DVar as an extra proposal density in an EPFM, known as hybrid ensemble and variational DA framework for environmental systems method (HEAVEN; Abbaszadeh et al., 2019).

The evolutional swarm‐like PFs contain several steps and assumptions for mutation and cross‐over without guaranteeing convergence to the full posterior PDF in the limit of an infinite ensemble size. Less significant approximations are needed in the Evolutionary PF‐MCMC (EPFM) method described in Abbaszadeh et al. (2018) where GA‐MCMC is used to define the importance sampling step. EPFM outperforms the PF‐MCMC providing more accurate and reliable results and overcomes the limitations of the recent standard PF‐GA algorithm where parameters of crossover and mutation steps need to be tuned. The EPFM method uses crossover and mutation step to generate new proposal model states. The crossover step consists in a linear combination of parent particles. The mutation process is carried out to increase the diversity among the particles. Afterward, the proposal particles are further refined with the MCMC approach. A Gaussian distribution of the proposal state is assumed to calculate metropolis acceptance ratio in the MCMC step. The HEAVEN (Abbaszadeh et al., 2019) integrates the EPFM algorithm and the 4D‐VAR to also account for model structure uncertainty other than model parameters and input uncertainties. Abbaszadeh et al. (2019) show that HEAVEN outperforms EPFM and better simulates streamflow in high flow regimes.

In this study, we adopt and evaluate an enhanced PF following the results of the previous studies by Di Mauro et al. (2021) and Hostache et al. (2018). The DA approach, hereafter called tempered particle filter (TPF), applies tempering coefficients to inflate the likelihood within an iterative process so that the Bayes' formula is respected (Beskos et al., 2014). The method is based on the method first proposed by R. M. Neal (1996), combined with ideas from Herbst and Schorfheide (2019). The iterative assimilation approach is based on successive Sequential Importance Resamplings (SIRs) and particle mutations (Abbaszadeh et al., 2018; Han et al., 2011; Li et al., 2005; Moradkhani et al., 2005). The mutations enable the ensemble to regain diversity after each resampling step in each iteration and are based on a Metropolis Hasting (MH) algorithm. We hypothesize that the proposed DA methodology enables the mitigation of some PF limitations, sample degeneracy, and sample impoverishment, while preserving the assimilation performances in terms of flood extent, discharge, and water level simulations.

In this study, we also further investigate additional benefits that come from this new approach. According to Dasgupta et al. (2021), degeneracy plays a crucial role in the persistence of the assimilation benefits over several time steps. Therefore the TPF approach could also help with improving the persistence of the assimilation benefits. Moreover, DA algorithms often assume that the observations as well as the model predictions are unbiased. Many authors pointed out the importance of bias removal before the DA, but it is not a straightforward procedure, especially in model forecasts (De Lannoy et al., 2007). Bias can depend on the model structure or parameters, on the initial conditions, or on forcing errors (especially when the forcings are derived from a forecast model, as in this study). In this context, we hypothesize that the new approach based on a TPF enables the reduction of bias in the model predictions and we test this hypothesis. To enable a meaningful evaluation and to verify whether the new approach outperforms the previous one, the TPF performance is compared to that of the SIS.

We carry out twin experiments based on a synthetically generated data set with controlled uncertainty. The SAR observations are synthetically generated from the simulated flood extent maps and assimilated into a coupled hydrologic‐hydraulic model. Two different background ensembles, that is, Open Loops (OLs), are drawn and used: in the first case, the ensemble encompasses the synthetic truth most of the time, in the second case the ensemble is most of the time outside the ensemble range.

The objectives of this study are therefore (a) to evaluate whether a principled method, in which the only approximation is the finite ensemble size, can mitigate degeneracy, (b) to evaluate whether the proposed framework improves the prediction accuracy and increases the persistence of the assimilation benefits, (c) to evaluate the efficiency of the method in reducing forecast bias. The paper is structured as follows: Section 2 describes the materials and methods, Section 3 showcases and discusses the results and three draws the conclusions of the study.

## 2 Materials and Methods

The first part of this section presents the structure of the flood forecasting system. The second part describes the proposed assimilation framework based on a TPF. The experimental design, case study, and the performance metrics used within this experiment are introduced in the last part.

### 2.1 The Flood Forecasting Model

**Figure 1:**
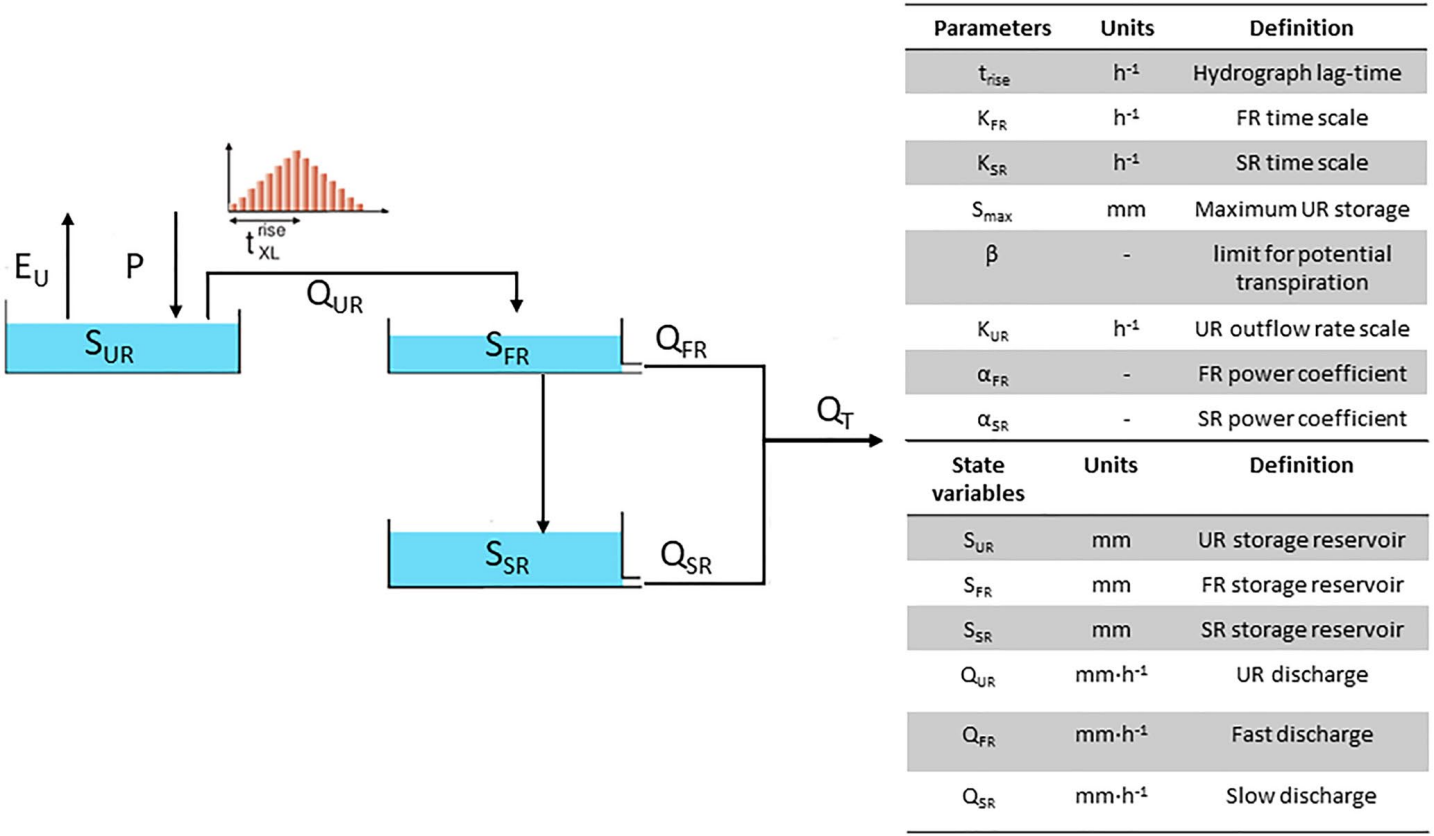
Scheme of the SUPERFLEX model used in this study. The hydrological model is based on three reservoirs: an unsaturated soil reservoir (S_*UR*_), a fast run‐off reservoir (S_*FR*_), and a slow run‐off reservoir (S_*SR*_). The discharge deriving from the three reservoirs are: Q_*UR*_, Q_*FR*_, Q_*SR*_. A triangular lag function with a base length equal to 2 ⋅ *t* ^rise^ is applied at the outflow of the unsaturated soil reservoir. E_*U*_ and P represent the potential evaporation and rainfall respectively.

We use the ERA5 data set (Hersbach et al., 2019) to derive the forcing of the flood forecasting system. Rainfall and 2 m air temperature at a spatial resolution of approximately 25 km and a temporal resolution of 1 hr are used as inputs to the flood forecasting system. A conceptual hydrological modeling framework (SUPERFLEX) coupled with a hydraulic model (LISFLOOD‐FP) approach has been adopted: the run‐off estimated with the hydrological model is used as input to the shallow water hydraulic model. In this study, the rainfall‐runoff model SUPERFLEX (Fenicia et al., 2011) is a lumped conceptual model. The state variables and the parameters used are listed in Figure 1. The conceptualization model is composed of three reservoirs: an unsaturated soil reservoir with a storage *S*_*UR*_ representing the root zone, a fast reservoir with storage *S*_*FR*_ representing the fast responding components (e.g., the riparian zone and preferential flow paths), and a slow reservoir with storage *S*_*SR*_ representing slow responding components (e.g., deep groundwater). A lag function is used at the outlet of the unsaturated soil reservoir to enable a delayed hydrological response of the basin under intense rainfall conditions. The hydraulic model is based on LISFLOOD‐FP (Bates & Roo, 2000; J. Neal et al., 2012) and simulates flood extent, water level, and discharge within the hydraulic model domain. The roughness coefficient and the bathymetry of the hydraulic model have been previously calibrated (Wood et al., 2016).

ERA5 rainfall time series are used to generate the synthetic truth and are also perturbed to generate an OL simulations consisting in 32 particles. These 32 particles are then used as input to the flood forecasting model to obtain the ensemble of flood extent maps. We adopt the method proposed and detailed in Di Mauro et al. (2021) to generate synthetic observations from model results. The flood extent map of the synthetic truth together with a real SAR observation are used to compute probabilistic flood maps (PFMs) where each pixel represents the probability to be flooded given the recorded backscatter values (Giustarini et al., 2016). During the analysis (i.e., assimilation) step, the generated PFMs are assimilated into the ensemble of wet‐dry maps via the TPF to obtain the updated particles. The following section describes the DA framework.

### 2.2 Data Assimilation Framework

PFs are based on Bayes' theorem:

(1) $$p\left(x^{k}|y^{k}\right)=\frac{p\left(y^{k}|x^{k}\right)}{p\left(y^{k}\right)}p\left(x^{k}\right)$$

The observation *y* at time *k*, which is the probability to be flooded given the SAR backscatter value, is combined with the forecasts of the numerical model *x* at time *k*. The posterior probability *p*(*x* ^*k*^∣*y* ^*k*^) is computed by multiplying the prior probability density function *p*(*x* ^*k*^), which is the probability of the model before any observation is taken into account, with the likelihood *p*(*y* ^*k*^∣*x* ^*k*^) that is the probability density that the model state *x* ^*n*^ produces the observation. In PFs, the prior PDF is drawn from an ensemble of model states of size *N* called particles. Equation 2 represents the computation of the prior probability:

(2) $$p\left(x^{k}\right)\approx \sum_{n=1}^{N}\frac{1}{N}\delta \left(x^{k}-x_{n}^{k}\right)$$

where *δ* is the Dirac delta function. Inserting Equation 2 into Equation 1 leads to the posterior probability formula:

(3) $$p\left(x^{k}|y^{k}\right)\approx \sum_{n=1}^{N}W_{n}\delta \left(x^{k}-x_{n}^{k}\right)\;where\;\;W_{n}=\frac{p\left(y^{k}|x^{k}\right)}{p\left(y^{k}\right)}$$

The weights *W*_*n*_, hereafter called global weights, were computed by the multiplication of the pixel‐based local weights $w_{i}^{n}$, according to the formula by Hostache et al. (2018), assuming that observation errors are independent across space. The set of particles tends to degenerate: after the assimilation, the number of particles with significant weight is reduced to a few and the posterior distribution is poorly approximated. Di Mauro et al. (2021) made a first attempt to reduce degeneracy, within this DA framework, using a tempering coefficient *γ* according to the formula:

(4) $$p\left(x^{k}|y^{k}\right)={\left(\frac{p\left(y^{k}|x^{k}\right)}{p\left(y^{k}\right)}\right)}^{\gamma }p\left(x^{k}\right)\;with\;\;\gamma \in \left[0,1\right]$$

This technical solution enables inflating the posterior variance so that several particles keep significant weight. However, it is an approximate solution as not all information from the observations is taken into account.

In the current study, we aim to further improve the application of the likelihood tempering. The proposed method relies on the factorization of the likelihood through an iterative approach according to the following formula:

(5) $$\frac{p\left(y^{k}|x^{k}\right)}{p\left(y^{k}\right)}=\prod_{s=1}^{S}{\left(\frac{p\left(y^{k}|x^{k}\right)}{p\left(y^{k}\right)}\right)}^{\gamma_{s}}$$

where 0 < *γ*_*s*_ < 1 for each iteration, *s* and $\sum_{s=1}^{S}\gamma_{s}=1$.

This factorization enables application of the Bayes' theorem iteratively so that the transition from the prior to the posterior probability is smoothly processed. The iterative methodology leads to the following equation after one iteration:

(6) $$p\left(y^{k}|x^{k}\right)=\prod_{s=2}^{S}{\left(\frac{p\left(y^{k}|x^{k}\right)}{p\left(y^{k}\right)}\right)}^{\gamma_{s}}{\left(\frac{p\left(y^{k}|x^{k}\right)}{p\left(y^{k}\right)}\right)}^{\gamma_{1}}p\left(x^{k}\right)$$

leading to:

(7) $$p\left(y^{k}|x^{k}\right)=\prod_{s=2}^{S}{\left(\frac{p\left(y^{k}|x^{k}\right)}{p\left(y^{k}\right)}\right)}^{\gamma_{s}}p_{1}\left(x^{k}|y^{k}\right)p\left(x^{k}\right)$$

and:

(8) $$p_{1}\left(x^{k}|y^{k}\right)\approx \sum_{n=1}^{N}W_{n}^{\left(1\right)}\delta \left(x^{k}-x_{n}^{k}\right)\;with\;\;W_{n}^{\left(1\right)}={\left(\frac{p\left(y^{k}|x^{k}\right)}{p\left(y^{k}\right)}\right)}^{\gamma_{1}}$$

At each iteration *s*, the tempering coefficient *γ*_*s*_ enables inflation of the likelihood variance and reduction of the weight variance, therefore reducing degeneracy. The exponent *γ*_*s*_ allows to keep a substantial number of particles with significant weights. At each iteration *s*, the *γ*_*s*_ value is increased and represents the solution to Equation 9:

(9) $$\mathrm{In}\mathrm{Eff}\left(\gamma_{s}\right)=r^{*}$$

where the ensemble inefficiency ratio (*InEff*) is given by Equation 10:

(10) $$\mathrm{In}\mathrm{Eff}\left(\gamma_{s}\right)=\frac{1}{N}\sum_{n=1}^{N}{\left(W_{n}^{s}\left(\gamma_{s}\right)\right)}^{2}\;$$

and a target value r* of the *InEff* is previously defined. Iterations are stopped when *InEff*(1) < r* and $\gamma_{S}=1-\sum_{s=1}^{S-1}\gamma_{s}$, where *S* is the total number of iterations.

After each iteration *s*, the particles with high weights are resampled using the SIR algorithm proposed by Gordon et al. (1993). Particles are replicated proportionally with their weights: those with an associated low importance weight are replaced with replicas of those having higher weight. After resampling, particles are equally weighted.

Next, a mutation is applied to the fast run‐off reservoir level (S_*FR*_), a variable of the hydrological model, 24 hr prior to the assimilation to regain diversity within the particle ensemble and the mutated value is used as initial condition for a subsequent model simulation over the 24 hr preceding the assimilation time. Mutating the hydrological state variable 24 hr prior to the assimilation time and carrying out the related model simulations is done in order to update the hydrological and hydraulic models more consistently since the water depths simulated by the hydraulic model at a certain time are the result not only of the current but also of the past upstream streamflow conditions.

This mutation is carried out using an MH algorithm, based on a random perturbation via the steps of MCMC methods. Since the model is deterministic a mutation of the state 24 hr back in time leads to a corresponding unique mutation at present time. This allows us to write $p\left(y^{k}|x_{j}^{k}\right)=p\left(y^{k}|x_{j}^{k-1}\right)$ for each particle *j*. Hence, the MH is based on two steps: first, draw a new particle from a proposal density as $x^{*}∼q\left(x|x_{j}^{k-1}\right)$, and then calculate the MH acceptance ratio:

(11) $$\alpha =\min\left\{1,\left(\frac{p\left(y^{k}|x^{*}\right)\;p\left(x^{*}\right)}{p\left(y^{k}|x_{j}^{k-1}\right)\;p\left(x_{j}^{k-1}\right)}\frac{q\left(x_{j}^{k-1}|x^{*}\right)}{q\left(x^{*}|x_{j}^{k-1}\right)}\right)\right\}$$

Many possibilities are available for choosing the proposal density *q*(). As detailed below, we choose it symmetric in order to cancel the proposal density ratio. Furthermore, since the prior 24 hr back is much wider than the likelihood, we can safely ignore the ratio $p\left(x^{*}\right)/p\left(x_{j}^{k-1}\right)$, and in this case the acceptance ratio becomes:

(12) $$\alpha =\min\left\{1,\left(\frac{p\left(y^{k}|x^{*}\right)}{p\left(y^{k}|x_{j}^{k}\right)}\right)\right\}$$

where $x_{j}^{k}$ represents the particles with high weight that have been resampled. A random variable *u* ∼ *U*[0, 1] is drawn and the mutated particle is accepted if *α* > *u*, otherwise we keep the particle as before its mutation.

As proposed by Herbst and Schorfheide (2019), the mutation is carried out based on a proposed innovation $p\left(x^{*}|x^{k-1}\right)=N\left(x^{k-1},c_{s}^{2}\cdot \sigma^{2}\right)$, with *c*_*s*_ being a scaling factor given by the following equation:

(13) $$c_{s}=c_{s-1}\left(0.95+0.10\cdot \frac{e^{20\cdot \left(\alpha -0.4\right)}}{1+e^{20\cdot \left(\alpha -0.4\right)}}\right)$$

*c*_*s*_ at the first iteration is set to 0.2. The mutation step is repeated for *l* = 1, .., *N* ^*MH*^. In our study *N* ^*MH*^ = 2.

**Figure 2:**
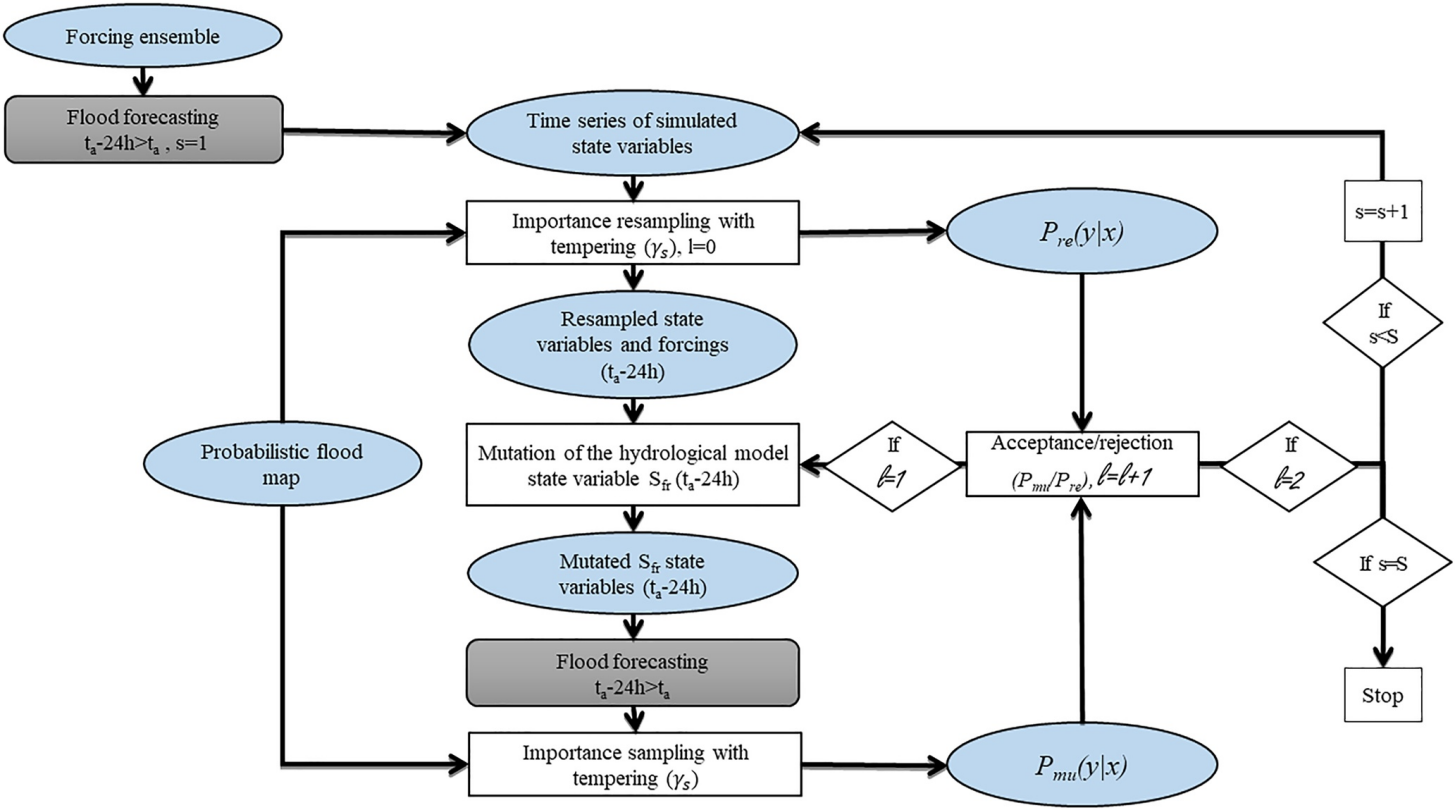
Flow chart of the data assimilation framework where synthetic probabilistic flood maps are generated from flood extents, derived from a truth run, and assimilated within the same flood forecasting model. The flood forecasting model is represented with a gray rectangle, mathematical operations with a white rectangle, state variables, input, and observations with a blue ellipse.

In detail, the method is structured according to the following time steps (Figure 2):

1. Ensemble forcing are used as input to the flood forecasting model;
2. The hydrodynamic simulations are carried out over the 24 hr prior to the assimilation.
3. Calculate *p*(*y*|*x*_*i*_) for each particle *i* and find *γ*_1_ such that *InEff*(1) ≥ r*.
4. Particles are resampled using the tempered weights. The particles after resampling that are duplicates of particles with high weights are perturbed at time *t*_*a*_‐24 hr.
5. New hydrodynamic simulations with the mutated levels of the S_*FR*_ are carried out during the 24 hr prior to the assimilation.
6. The likelihood of the mutated particles *p*_*mu*_(*y*∣*x*) is compared to the likelihood of the resampled particles *p*_*re*_(*y*∣*x*).
7. The resampled particles are replaced by the mutated particles if the ratio of the two is larger than a value randomly taken from the interval [0, 1].
8. The mutation step is repeated twice.
9. The iteration with a new tempering coefficient is realized.
10. The entire process is repeated until the sum of the tempering coefficients is equal to unity.

### 2.3 Experimental Design, Case Study, and Performance Metrics

**Figure 3:**
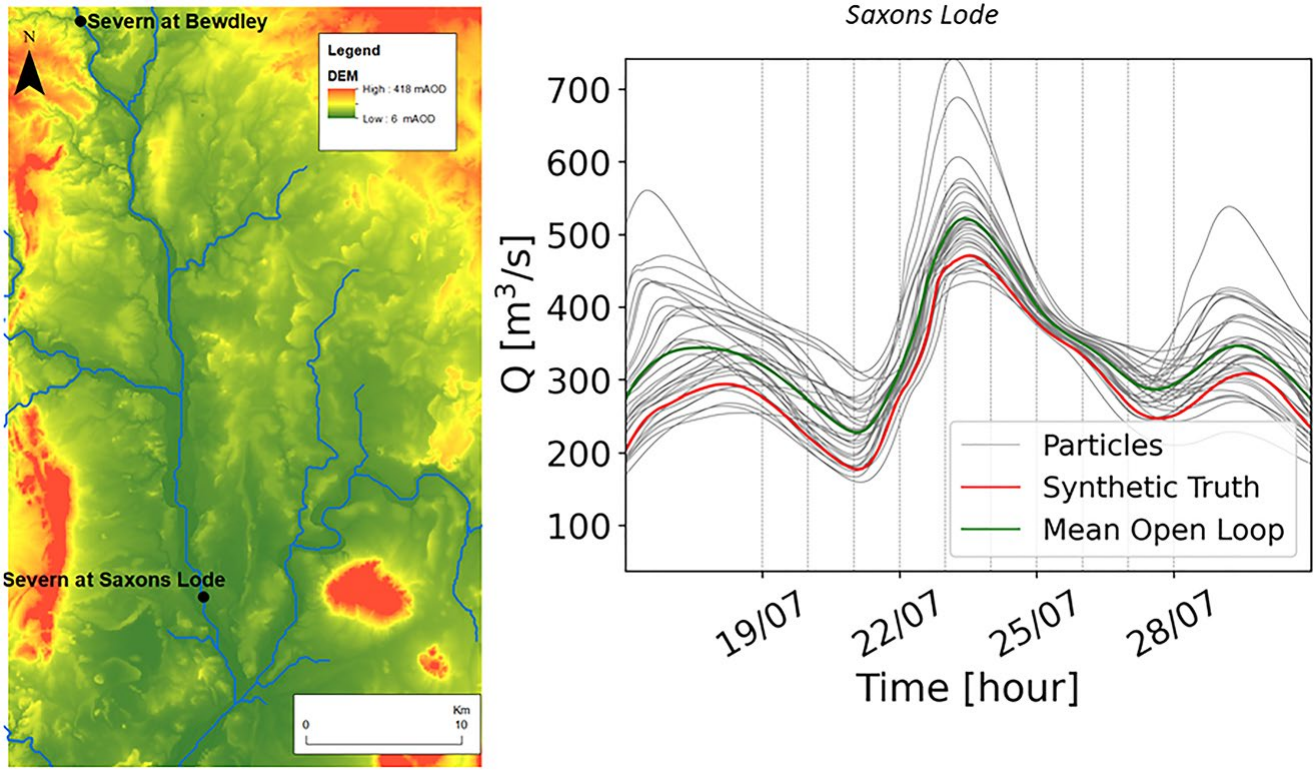
Study area of the synthetic experiment (left). Black dots correspond to the points where evaluation of the data assimilation performances is carried out (“Severn at Bewdley” and “Severn at Saxons Lode”). Ensemble time series of discharge in Saxons Lode and assimilation times (right). Gray lines correspond to the Open Loop (OL), the red line corresponds to the synthetic truth, the green line corresponds to the mean of the OL. The dashed lines correspond to the different assimilation time steps performed independently every 24 hr from 19 July 00:00 to 28 July 00:00.

The study area is the lower river Severn located in the United Kingdom (Figure 3, on the left). To analyze the filter performances at different assimilation times, SAR images have been synthetically generated (see Di Mauro et al., 2021) every 24 hr from 19 July 00:00 to 28 July 00:00 (Figure 3, on the right) and the 10 corresponding independent assimilations are carried out and evaluated.

The flood event has been simulated using the rainfall and temperature (ERA‐5 data set) time series corresponding to the July 2007 event as input data to the flood forecasting system.

Further details concerning the hydrological and hydraulic model set‐up as well as the study area of the synthetic experiment, are provided in our previous study (Di Mauro et al., 2021). In this study, the ensemble contains 32 particles. The proposed TPF is characterized by a particle mutation at each iteration. The mutation step could have a key‐role, especially when the ensemble is biased with respect to the observations. On the one hand, in the SIS case, the weighted mean (also called expectation) is based on the initial particles of the ensemble meaning that if the truth falls outside the ensemble range the expectation cannot reach the synthetic truth. On the other hand, in the TPF case, the particles can mutate and move outside the initial ensemble range. This way the expectation can potentially reach the synthetic truth. For evaluating the capability of the TPF to compensate for bias within the ensemble, two different cases are investigated. The difference between the OL and the synthetic truth (O) rainfall time series averaged over the flood event period (K) represents the mean bias error (MBE, Equation 14) and it is used to estimate the bias. For a “markedly” biased case MBE is 0.92 $\frac{mm}{h}$ while for a “limited” bias case the MBE is 0.14 $\frac{mm}{h}$, meaning that the error of the markedly biased case is 6.56 times larger than for the other case.

(14) $$\mathrm{MBE}=\frac{1}{K}\sum_{k=1}^{K}\left(OL_{k}-O_{k}\right)$$

In the limited case, the synthetic truth is most of the time within the ensemble range; in the other case the ensemble is conspicuously biased and the synthetic truth falls outside the ensemble range most of the time. The assimilation steps are performed at the same time for both cases and the same observations are used.

Results are analyzed according to different spatial (global and local) and temporal scales (at the assimilation time and for the subsequent time steps). The filter performances are evaluated in terms of predicted flood extent and water depth maps, as well as local discharge and water levels time series. The performance metrics are assessed by comparing the results of the TPF with those of the OL. Moreover, the TPF is compared with the SIS method applied in our previous study Di Mauro et al. (2021). The local evaluation of the prediction accuracy of water levels and discharge is performed by comparing the simulated discharge and water level time series with respect to the synthetic truth.

The following performance metrics are used:

1. Confusion matrices: a matrix providing the number of false negatives (under‐prediction) and false positives (over‐prediction), together with correct positives and negatives;
2. Contingency maps: maps comparing the simulated flood map with the synthetic truth map;
3. Critical success index (CSI): a metric that evaluates the accuracy of the flood map predictions and is defined as the ratio between the number of pixels correctly predicted as flooded over the sum of predicted flooded pixels (correct positives, false positives, and false negatives). It ranges from 0, complete disagreement, to 1, perfect match;
4. Root mean square error (RMSE): it is given by the square root of the mean of the squares of the deviations of the predicted water levels against the synthetic truth over the hydraulic model domain. It evaluates the prediction errors of a state variable, in our case the water levels.
5. 95% Exceedance Ratio (ER_95_): it measures the reliability of the ensemble prediction quantiles and it is given by the formula: (*N*_exceedence_/*T*) ⋅ 100, where *N*_exceedence_ is the number of times during the total simulation *T* where observations fall outside the 95% predictive bounds. The ideal ensemble should fall outside the 95% predictive bounds only the 5% of the time (Moradkhani et al., 2006).
6. Normalized RMSE ratio (NRR): it is a normalized measure of the ensemble dispersion. It is defined as the ratio of the time‐averaged RMSE of the ensemble mean to the time‐averaged RMSE of the single members of the ensemble over the value $\sqrt{\left(N+1\right)/2N}$ and it should be equal to one. NRR > 1 indicates an insufficient spread, while NRR < 1 indicates the opposite (Anderson, 2001; Moradkhani et al., 2005).

## 3 Results and Discussions

### 3.1 TPF‐Based Assimilation Performances

#### 3.1.1 Flood Extent Map Predictions

**Figure 4:**
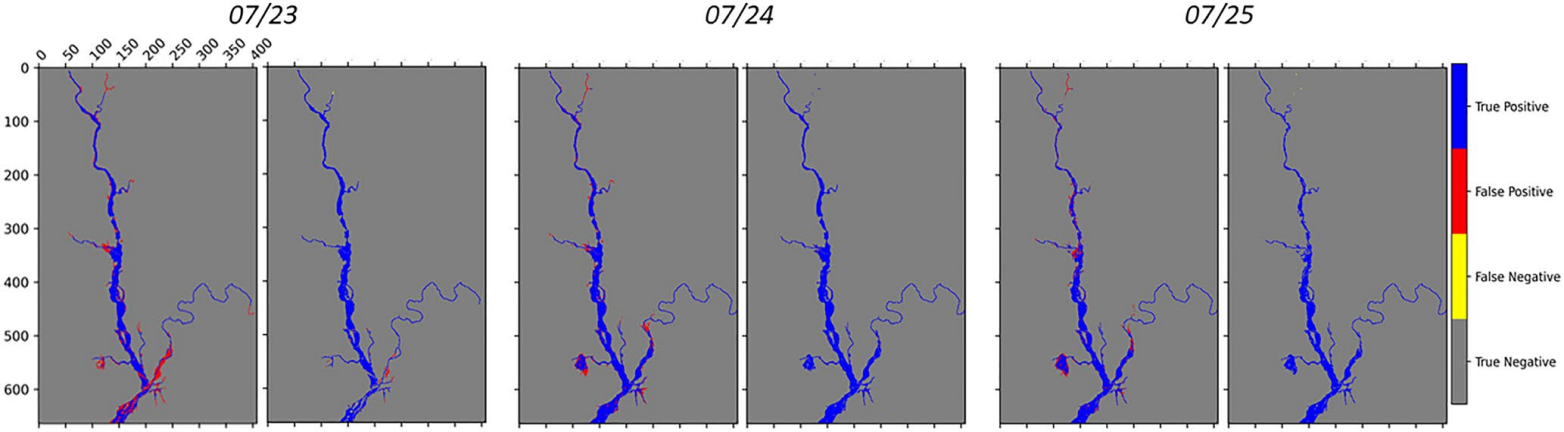
Contingency maps of the Open Loop (left) and after the assimilation (right) for three different assimilations at time 23 July 00:00, 24 July 00:00, 25 July 00:00. Red pixels correspond to over‐prediction (false positives) errors, yellow pixels to under‐prediction (false negatives) errors, pixels correctly classified as not‐flooded are in gray and when the contrary occurs pixels are in blue.

The flood extent maps are evaluated via different performance metrics: the contingency maps, the CSI and the confusion matrix. The contingency map is derived from the comparison between the simulated flood extent map (i.e., expectation) and the validation map which is derived from the synthetic truth simulation in our case. The contingency maps, corresponding to three different assimilation time steps (rising limb, peak, falling limb), are shown in Figure 4.

Yellow and red pixels correspond to errors of under‐prediction (when the model wrongly predicts the pixels as not‐flooded) and over‐prediction (the opposite case), respectively. In Figure 4, the reported images for each assimilation time correspond to the OL (on the left) and the TPF analysis (on the right). Over‐prediction represents the most frequent type of error and it is significantly reduced as a result of the TPF‐based assimilation.

The decrease of wrongly predicted pixels is quantified in the confusion matrix reported in Table 1. In line with Figure 4, after any of the three assimilation time steps, the number of over‐prediction errors is reduced by 90% or more, while the number of under‐predicted pixels increases in the upstream part of the river. However, they represent only 0.3% or less of the total number of flooded pixels.

**Table 1:** Confusion Matrix of the Open Loop and Tempered Particle Filter Analysis for Three Different Time Steps (23 July 00:00, 24 July 00:00, 25 July 00:00)

Method		23 July 00:00		24 July 00:00		25 July 00:00	
		PF	PN	PF	PN	PF	PN
Open	TF	7,497	0	9,374	0	8,390	1
Loop	TN	2,441	260,974	1,356	260,182	1,219	261,302
TPF	TF	7,475	22	9,374	22	8,378	13
	TN	204	263,211	78	261,460	30	262,491

*Note*. TF, flooded pixels in the truth map, TN, not‐flooded pixels in the truth map, PF, predicted flooded pixels, PN, predicted non‐flooded pixels.

**Figure 5:**
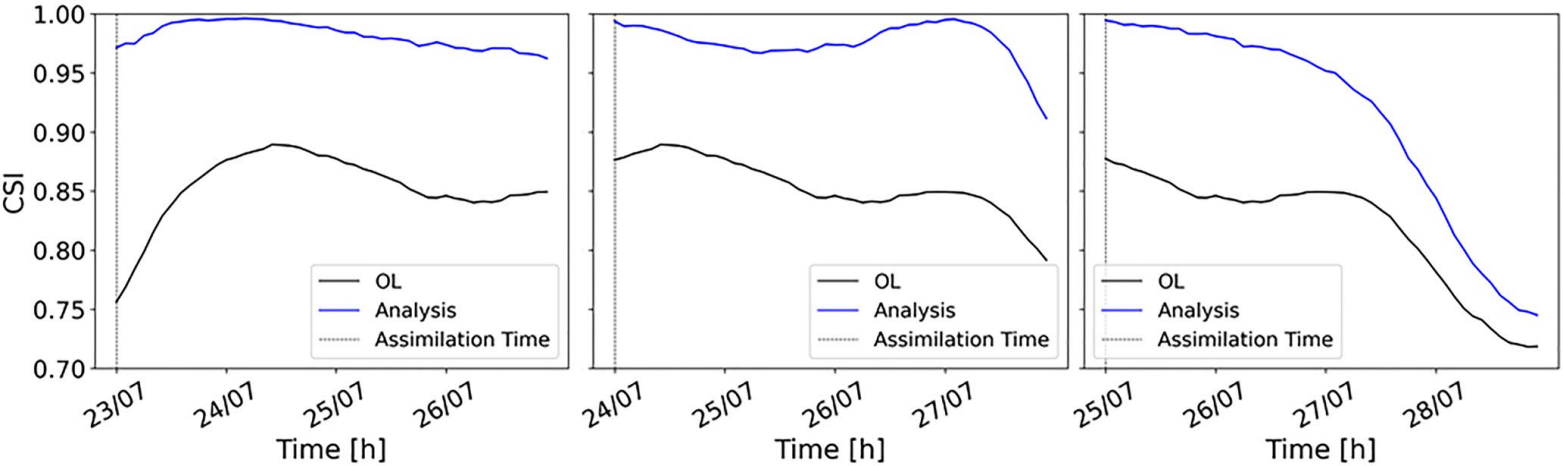
Hourly time series of the critical success index of the Open Loop (black line) and tempered particle filter analysis (blue line) due to the assimilation of three different images: during the rising limb (23 July 00:00), at the peak (24 July 00:00) and during the falling limb (25 July 00:00).

Time series of CSI are also used to evaluate the TPF performances (Figure 5). They allow to evaluate the predicted flood extent maps not only at the assimilation time step (as for the contingency maps and the confusion matrices) but also for subsequent time steps. Time series of CSI provide an assessment of the persistence of the improvements over longer lead times after the assimilation. Figure 5 shows the time series of CSI before (black line) and after (blue line) the assimilation of SAR images taken during the rising limb (23 July 00:00), at the peak (24 July 00:00) and during the falling limb (25 July 00:00) of the flood event.

This figure shows an improvement of the analysis compared to the OL not only at the assimilation time but also over subsequent time steps: on average, CSI improvements persist for more than 3 days after the TPF application.

#### 3.1.2 Water Level and Discharge Predictions

**Figure 6:**
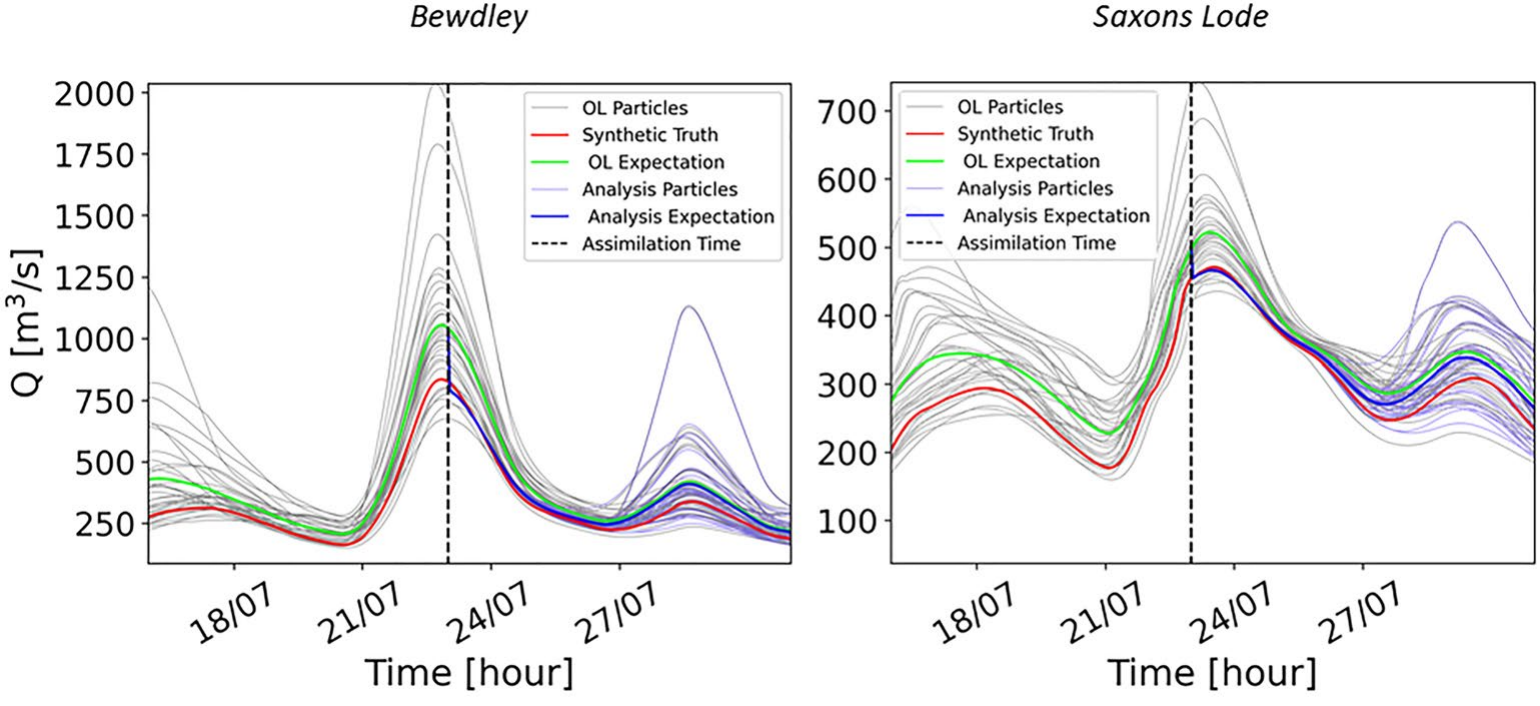
Time series of discharge at the peak at Bewdley and at Saxons Lode with the assimilation of an image at 23 July 00:00. The vertical dashed lines indicate the time of the assimilation. The gray lines correspond to the Open Loop (OL) particles, the green line to the OL mean, the light blue lines to the analysis particles and the blue line to the analysis expectation. The synthetic truth is represented by a red line.

To further investigate the TPF assimilation performance we evaluate water level and discharge predictions. This evaluation is carried out first at specific points along the river Severn: in Bewdley (the gauge station located at the upstream boundary of the hydraulic model domain), and in Saxons Lode (within the hydraulic domain). In Figure 6, the discharge at Bewdley (on the left) and at Saxons Lode (on the right) are plotted. The analysis expectation of discharge (blue line) moves closer to the synthetic truth (red line) at the two stations as a result of the assimilation showing a substantial improvement of the predictions. Here, we show the results from the assimilation on 23 July 00:00 as an illustrative example since the other assimilations produce similar effects. In Figure 6, it can be observed that the degeneracy is mitigated. At the assimilation time, the analysis particles are very similar and close to the synthetic truth, but rapidly regain diversity, thereby avoiding degeneracy. After more than 3 days, the particles return to their initial trajectories (i.e., the OL) mainly because precipitation uncertainty seems to prevail in the forecasts from that moment on.

**Figure 7:**
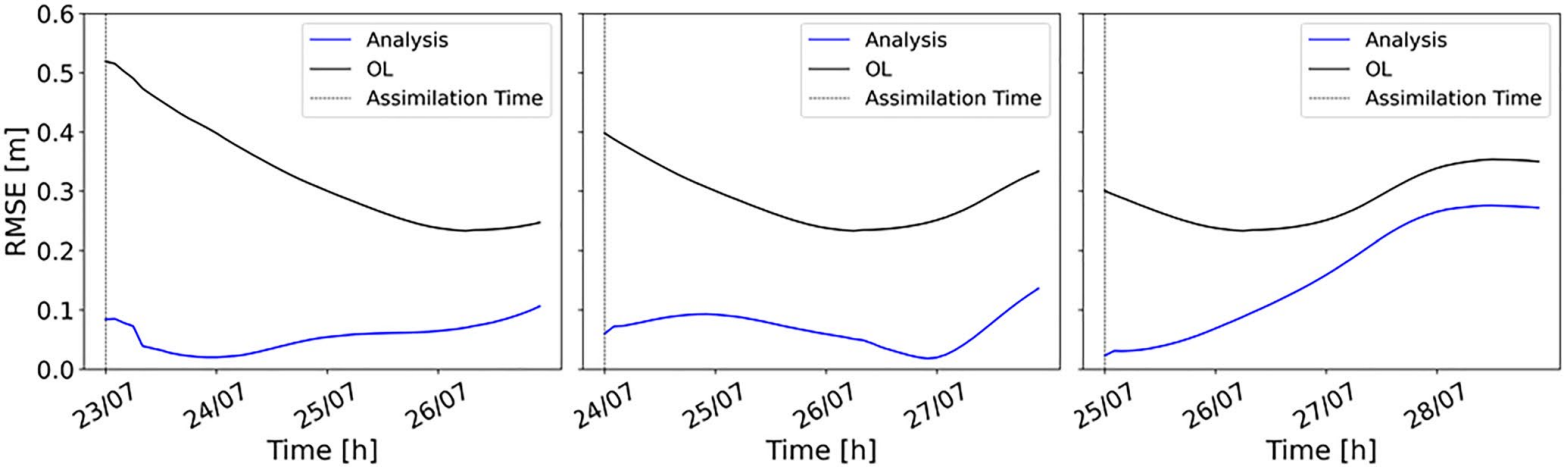
Hourly time series of the root mean square error. Black line refers to the Open Loop (OL) and blue line to the analysis results after the assimilations of three different images (23 July 00:00, 24 July 00:00, and 25 July 00:00).

**Figure 8:**
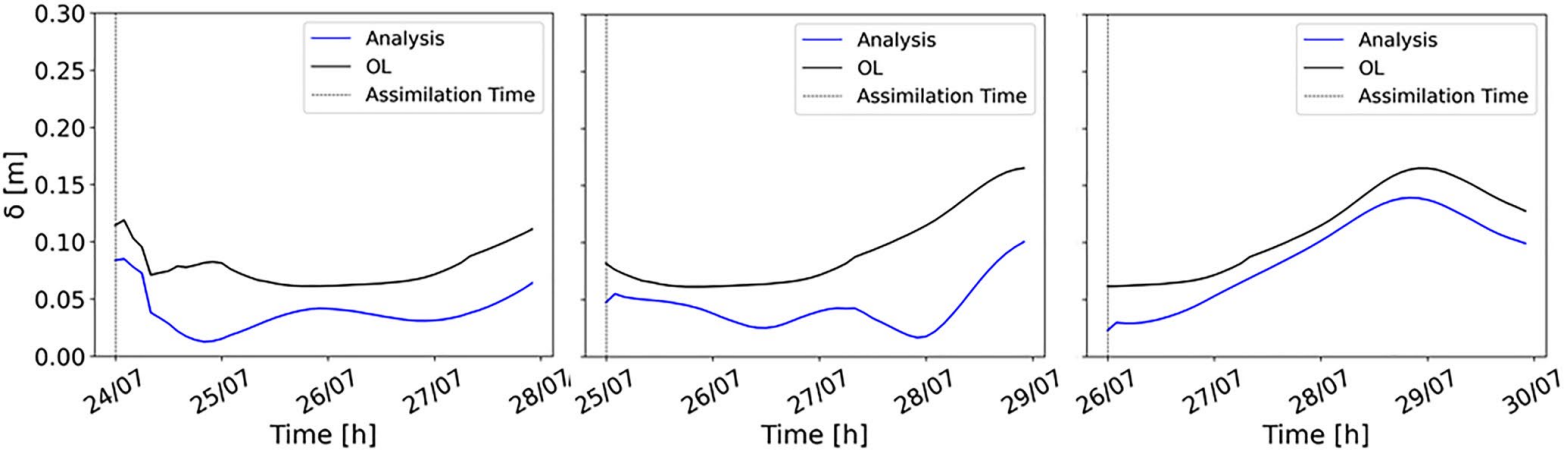
Hourly time series of the standard deviation of the errors due to the assimilation of three different images: 24 July 00:00, 25 July 00:00, and 26 July 00:00. The standard deviation of the errors as difference between the Open Loop (OL) and the true water levels (black line) and as difference between the analysis expectation and the true water levels (blue line).

To generalize the evaluation made for the gauging stations, we evaluate the accuracy of water level predictions globally, using time series of RMSE computed over the entire hydraulic model domain. This index has been calculated at the assimilation time and for subsequent time steps, in order to assess if the assimilation benefits persist in time. In Figure 7, the RMSE of the analysis is lower than the OL and this improvement lasts for more than 3 days following the assimilation. The accuracy of the results is higher when assimilation is performed after the flood peak, when rainfall has stopped, and inflow errors are dominating. Flood extents during the falling limb become more sensitive to changes in water depth due to the connectivity between the river channel and its floodplain (Dasgupta et al., 2021). Because of this high sensitivity, during the falling limb, flood extents change faster and weights should be updated more frequently to be consistent with the new hydraulic conditions. This could explain the reason why, as for the CSI plots (Figure 5), DA performances start dropping more quickly for the assimilation at the falling limb. The performances of the TPF experiment have been compared to those of the OL for lead time up to 7 days. After 1 week, we observe that the TPF‐CSI is 10% greater than the OL‐CSI whereas the TPF‐RMSE is 20% lower than the OL‐RMSE. These results show that the TPF still outperforms the OL after 1 week. The standard deviation of the errors has also been computed in order to evaluate the accuracy of the second moment (Figure 8). In this case, the standard deviation represents the dispersion of the errors (given as the difference between the expectation and the true water levels). Results show that the TPF application determines less dispersed and more clustered results around the synthetic truth.

### 3.2 Comparison Between TPF‐ and SIS‐Based Assimilation Experiments With Unbiased Background

**Figure 9:**
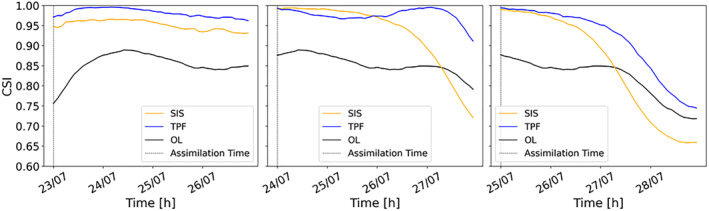
Comparison of the hourly time series of the critical success index of the Open Loop (OL) (black line), tempered particle filter (TPF) analysis (blue line) and Sequential Importance Sampling (SIS) analysis (orange line) due to the assimilation of three different images: 23 July 00:00, 24 July 00:00, and 25 July 00:00.

We showed in Section 3.1 that the TPF improves the predictions of water levels and discharge, as well as flood extent. In this section, the new TPF‐based DA framework is compared with the SIS approach previously proposed by Di Mauro et al. (2021). To do so, we apply the SIS method as proposed in Di Mauro et al. (2021) on the same 32 background particles (i.e., OL) and the same synthetically generated flood extent observations. The choice of comparing the TPF with this SIS is related to the fact that other methods reported in Di Mauro et al. (2021) were providing comparable performances, and therefore, SIS has been chosen as a benchmark. In terms of flood extent, the comparison is realized using the hourly time series of the CSI index (Figure 9).

In Figure 9, the blue line corresponds to the CSI of the forecast obtained from the TPF‐based case, the orange line to the one obtained from the SIS‐based case and the black line to the one of the OL. The three plots correspond respectively to the assimilation on 23 July 00:00, 24 July 00:00, and 25 July 00:00. The CSI values obtained when assimilating an image during the rising limb are systematically higher for the TPF. When the image is assimilated close to the peak and during the falling limb, CSI values of the TPF and SIS‐based assimilation are very similar at the assimilation time and for subsequent time steps. After 2 days, the performance of the SIS becomes substantially worse than that of the TPF. SIS suffers from degeneracy, the number of particles with a significant weight as a result of the assimilation is very limited. These particles produce accurate results at the assimilation time, but are not necessarily efficient after a few hours or days, especially when hydraulic conditions have changed in the meantime.

**Figure 10:**
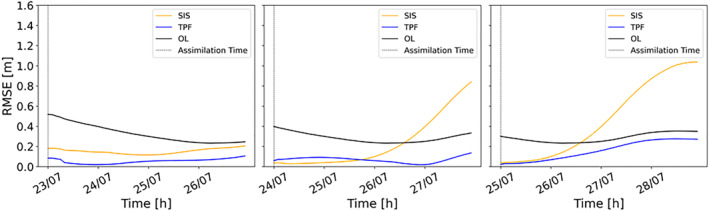
Hourly root mean square error (RMSE) time series. The black line represents the RMSE of the Open Loop (OL), the blue line the tempered particle filter (TPF)‐based RMSE and the orange line the SIS‐based RMSE. Three different assimilation cases are plotted: 23 July 00:00, 24 July 00:00, and 25 July 00:00.

We have also compared the performances of the SIS and the TPF using time series of RMSE (Figure 10). As expected, the RMSE time series exhibit very similar trend to the CSI: the RMSE is lower with the TPF experiment when assimilating an image during the rising limb. For the other two assimilation steps RMSE values are comparable, but performances of the SIS decrease more rapidly, especially after 2 days. Overall, Figures 9 and 10 clearly show the beneficial effects of the TPF assimilation on the long‐term.

Table 2 reports the ratios between the analysis‐RMSE and the OL‐RMSE for each assimilated SAR image and for different lead times. These ratios were calculated at each hour and for all the different assimilation dates. In the table, the values at the assimilation time and for lead times of 6 hr, 1 day, 2, 3, and 4 days are reported. The ratios obtained with the TPF method are shown in the gray cells. The cyan cells contain the ratios obtained with the SIS experiment. The last row of the table shows the mean of the RMSE ratios over the different assimilation times at given prediction lead times. The lower the RMSE ratio values, the better the performance. Ratios of RMSEs lower than unity indicate that the assimilation improves forecasts. Table 2 shows that the TPF‐based ratios are most of the time substantially lower than those of the SIS‐based ones. For instance, the SIS‐based mean ratios for 3 and 4 days of lead times are almost twice that of the TPF‐based one. The benefit of the TPF‐based assimilation persists for more than 4 days after the assimilation time. Moreover, the TPF‐based ratios are always lower than unity, whereas the SIS‐based ratios get also values higher than unity.

**Table 2:** Ratios Between the Analysis and Open Loop RMSE for Each Assimilation Date and for Various Lead Times

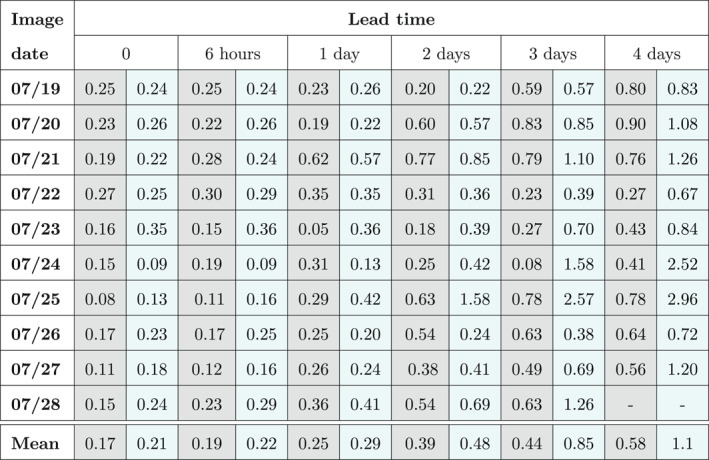

*Note.* Gray cells refer to the TPF‐based method, cyan cells to the SIS‐based method.

Model performances have also been statistically evaluated using the ER95 and the normalized root mean square error ratio (NRR). Both metrics have been used to evaluate the water level ensemble at two different gauge stations (Bewdley and Saxons Lode). ER_95_ evaluates the ensemble spread by quantifying the percentage of time the observation falls outside the 95% confidence interval derived from the ensemble. ER_95_ values should be ideally around 5%, meaning that the observation falls outside of the 95% predictive bounds only 5% of the time. NRR also evaluates the spread of the ensemble, ideal values should be around the unity and lower or higher values indicate a too narrow or too wide ensemble, respectively. Table 3 reports these statistical performances for the SIS and TPF experiments. While TPF‐ and SIS‐NRR are both close to the unity for the different assimilation time steps, ER_95_ varies with the different assimilation time steps. In particular, we found that on average, over the different assimilations, the value of ER_95_ for the TPF is around 7% in Bewdley and 9% in Saxons Lode, which are values close to the target values (5%). Moreover, if we compare these values with those of the SIS that are around 25%, it is clear that TPF substantially outperforms SIS. This highlights a marked degeneracy in the SIS, that is substantially reduced by TPF.

**Table 3:** Normalized Root Mean Square Error Ratio (NRR) and 95% Exceedance Ratio (ER_95_) of Water Levels at the Different Assimilation Times and at two Different Gauge Stations (Bewdley and Saxons Lode)

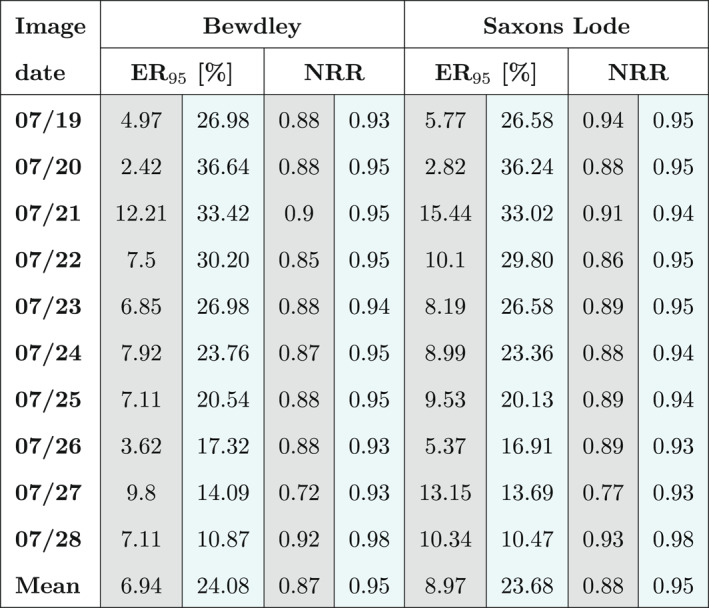

*Note.* SIS statistical performance measures are shown in the cyan column and TPF performance measures in the gray column. The average of the measures over the different assimilation time is also reported in the last row of the table.

### 3.3 Comparison Between TPF‐ and SIS‐Based Assimilation Experiments With Biased Background

**Figure 11:**
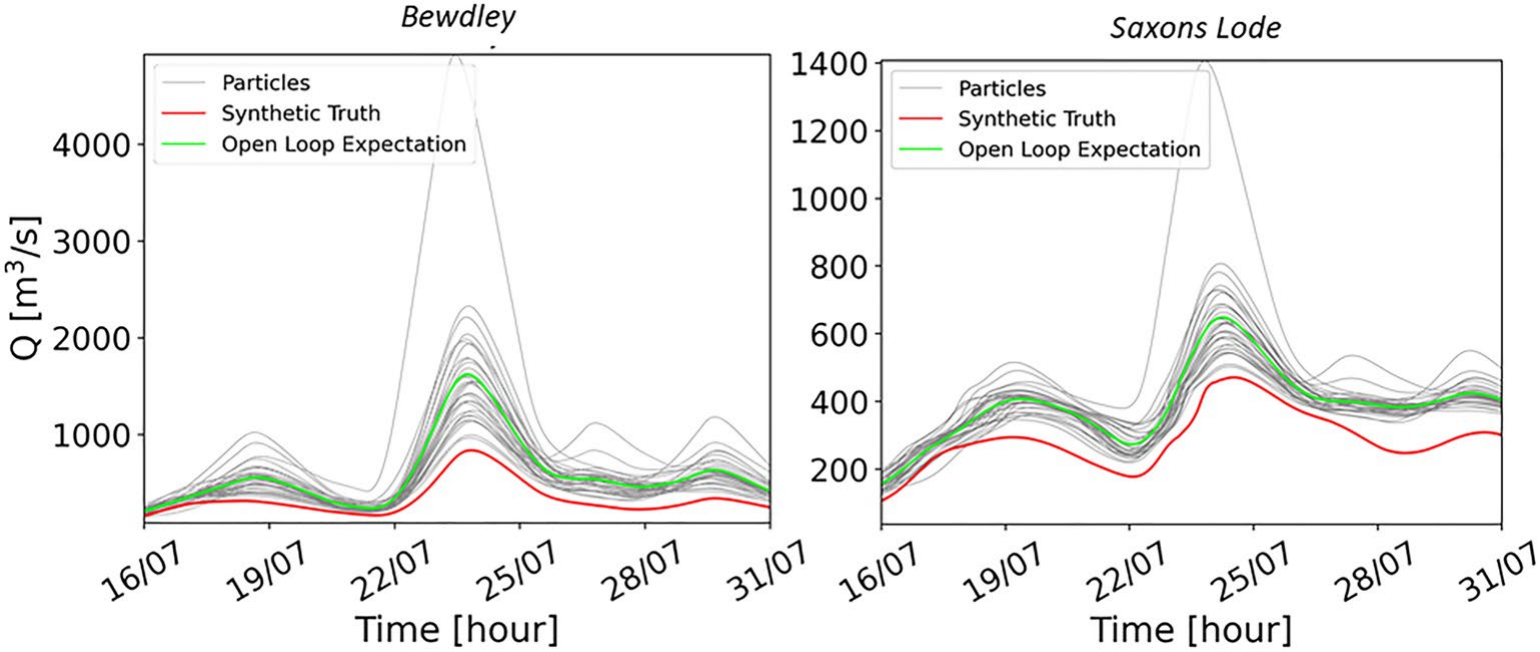
Discharge time series ensemble at Bewdley (on the left) and at Saxons Lode (on the right). The Open Loop (OL) particles are represented with gray lines, the synthetic truth is represented by the red line. The OL expectation is in green. In this case, the ensemble is markedly biased; the synthetic truth falls outside the ensemble range most of the time.

**Figure 12:**
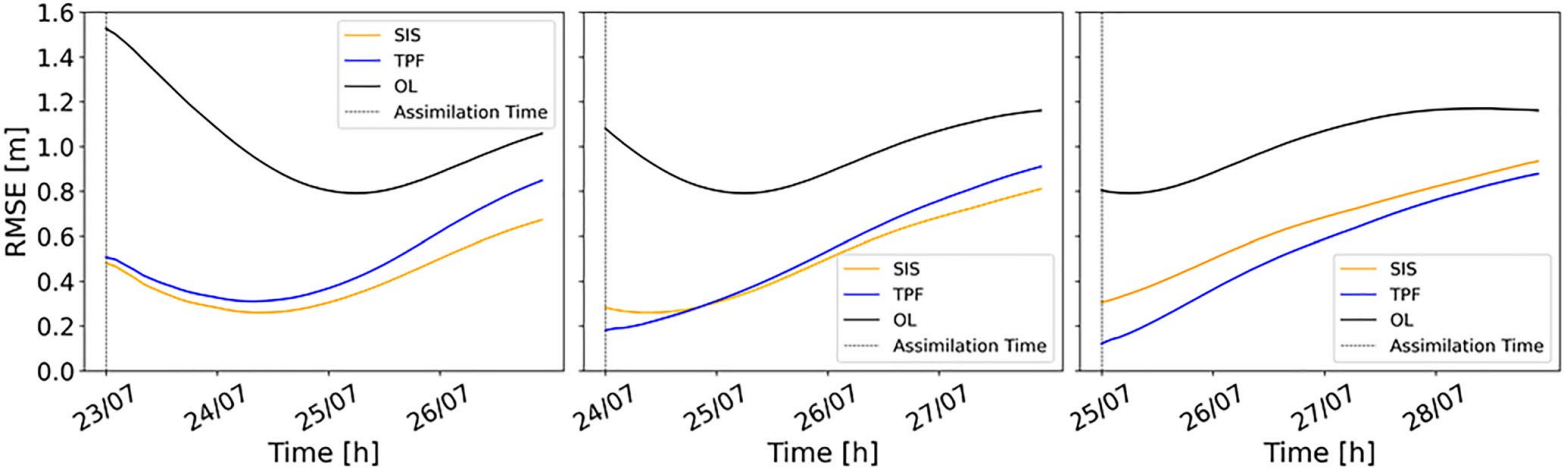
Hourly root mean square error (RMSE) time series for a markedly biased ensemble case. The black line represents the RMSE of the Open Loop (OL), the blue line the RMSE after the tempered particle filter (TPF) application and the orange line the RMSE after the Sequential Importance Sampling (SIS) application. Assimilation at 23 July 00:00, 24 July 00:00, and 25 July 00:00 are plotted.

In this last experiment, we use the same set‐up as in the previous experiment but with the exception of a modified OL. We have introduced a perturbation error to the ERA‐5 rainfall time series so that the bias in the ensemble is 6.56 times larger than in the previous case. The ensemble has significant bias and the synthetic truth is most of the time located outside of the ensemble range as can be seen in Figure 11. For the evaluation of the results, the same performance indices and the same plots are used. The ratios between the analysis‐RMSE and the OL‐RMSE for each assimilated SAR image and for different lead times are reported in Table 4. At the assimilation time and for more than 1 day after that, the TPF‐based assimilation is capable of substantially reducing the forecast bias. The SIS is less efficient in that respect, as RMSE ratios are larger for the SIS‐based assimilation. For longer lead times, the error in water levels increases due to the bias in the rainfall ensemble and the RMSE ratios of the TPF‐based and the SIS‐based assimilation become similar. This is clearly visible in Figure 12 which shows the RMSE time series on 23 July, 24 July, and 25 July at 00:00. When the bias is limited and the synthetic truth falls inside the ensemble range most of the time, as in the previous case (Figure 7), the forecast improvement lasts for longer lead times. However, when the ensemble is markedly biased (Figure 12), the TPF improves the results at the assimilation time but the level of improvement degrades more quickly compared to the limited biased case.

**Table 4:** Ratio Between the Analysis and Open Loop of the RMSE for Each Assimilation Date and for Various Lead Times for a Markedly Biased Case

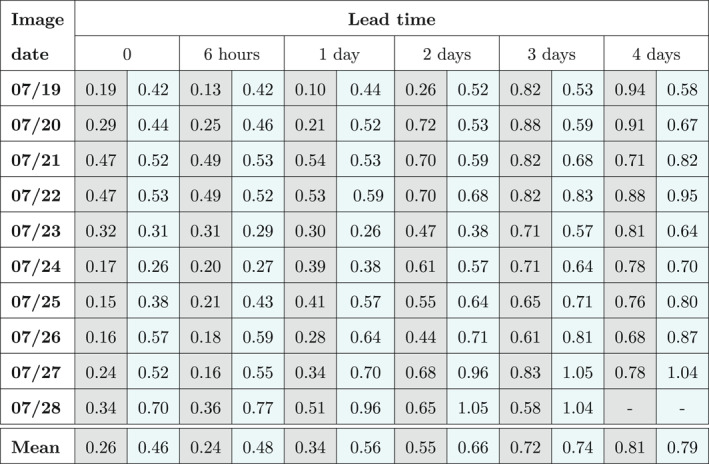

*Note.* Gray cells refer to the TPF‐based method, cyan cells to the SIS‐based method.

At the assimilation time, the TPF always improves the accuracy of the results of the flood forecasts (in terms of flood extent, water levels, discharge) with respect to the OL and it is comparable to the SIS performances. An important aspect that emerges from the results is the persistence of the assimilation benefits. They remain significant even 3 days after the TPF assimilation when compared to the SIS performances; nonetheless, performances start degrading with the onset of rainfall over the headwater catchment and rainfall uncertainty prevails in the forecast uncertainty. We argue that the marked improvement in the forecast skill of the TPF, compared to the SIS, is due to the update of the initial conditions of the hydrological model including S_*FR*_ 24 hr prior to the assimilation time. In the TPF, better initial conditions of the model forecast are defined at each assimilation time via the different iteration and mutation steps, whereas the SIS only defines the relative importance of each particle, without carrying out any better definition of the initial conditions of the model. The runoff that is used as upstream boundaries of the hydraulic model is a function of the storage S_*FR*_ of the hydrological model. Updating the S_*FR*_, and consequently the fast run‐off, represents an effective way to increase the long‐lasting effects of DA since runoff has the highest uncertainty deriving from poorly known rainfall as already pointed out by Matgen et al. (2010). This aspect, together with the mitigation of degeneracy, as hypothesized by Dasgupta et al. (2021), could explain the longer‐term persistence of DA benefits via the TPF.

After the TPF application, particles move toward the synthetic truth also in the case the truth falls outside the predictive bounds of the OL ensemble. Despite the improvements due to the TPF, performances are not as good as in the previous case. As a consequence, results obtained using the TPF are sometimes similar to those obtained using the SIS, or even slightly less satisfying when rainfall uncertainty dominates the system. The improvements resulting from the update of the initial conditions are vanished after a few days because of the bias in the ensemble and the model moves back to the OL state. The update of the state level of the reservoir has a time‐limited benefit. It is a state variable highly influenced by the inputs, and thus by the rainfall. In our experiment, the rainfall ensemble is obtained by perturbing the deterministic ERA‐5 product using a multiplicative noise. Therefore, when there is low‐intensity rainfall simulated in ERA‐5 the uncertainty is very limited. Moreover, as the rainfall ensemble is not updated, the ensemble analysis goes back to the OL trajectory after a while. This return of the analysis back to the OL is even more rapid when higher rainfall intensity is imposed to the model: the influence of the initial conditions is rapidly overruled by the forcing uncertainty. To increase the time window of the assimilation benefits, the update of hydrological model state variable could be completed by a forcing update or by a parameter update, as in Cooper et al. (2019) where channel friction is updated together with a state variable, but with the consequent risk of multiple acceptable solutions of the system according to the equifinality concept (Beven & Freer, 2001).

## 4 Conclusions

In this paper, we have proposed a new approach based on a TPF to assimilate flood extent maps into a flood forecasting system. The objective of this new DA framework is to mitigate degeneracy and sample impoverishment, well‐known issues in particle filtering. In the proposed TPF method, the number of tuning parameters is small with respect to methods such as PF‐MCMC (Andrieu et al., 2010; Moradkhani et al., 2012) thus rendering the TPF easily transferable to other situations. We also argue that this makes the approach potentially robust. Moreover, the TPF does not need cross‐over steps or assumptions on the prior PDF used in the MH acceptance ratio as in the Evolutionary PF‐MCMC (Abbaszadeh et al., 2018) or in the HEAVEN (Abbaszadeh et al., 2019). We have evaluated the performances of the filter in two different cases: with a limited forecast bias and with a more important forecast bias. The TPF has been compared against the standard PF, namely the SIS as used in previous studies (Di Mauro et al., 2021; Hostache et al., 2018). The following key conclusions are drawn from our experiments:

1. At the time of the assimilation, forecasts are very accurate locally: the forecast overlaps the synthetic truth for all the different assimilation cases and for both analyzed locations. Results are very satisfying at a larger scale as well: RMSE and CSI improve systematically as a result of the assimilation. On average, RMSE values decrease by 80% whereas CSI values increase by 30% as a result of the assimilation;
2. Results are also satisfying across time: the CSI and RMSE are improved up to 3 days after the assimilation;
3. Performances are improved compared to the OL and the SIS filter. The benefits of the newly introduced TPF‐based assimilation are longer persisting when compared to the effects obtained with assimilation techniques used in the previous studies;
4. The new assimilation framework significantly outperforms the SIS. SIS performance indices are generally comparable to the TPF ones at the assimilation time, but they tend to drop more rapidly, in general 2 days after the assimilation. For example, TPF‐based RMSE are 20% lower compared to the SIS‐based ones, 2 days after the assimilation;
5. When the ensemble is markedly biased results are significantly improved by the TPF at the assimilation times and for a few days after. Afterward, TPF and SIS based results are similar because the model state update cannot compensate for a too large bias in the precipitation ensemble.

The proposed DA framework based on a TPF holds promise for improving prediction accuracy for longer lead times. In this study, we have shown a synthetic experiment where rainfall and SAR observations are the only sources of uncertainty. In a future study, it will be interesting to apply and evaluate this enhanced approach on a real test case in a weakly controlled environment.

## Data Availability

The LISFLOOD‐FP model can be freely downloaded at http://www.bristol.ac.uk/geography/research/hydrology/models/lisflood. The river cross‐section data, the digital elevation model, and the gauging station water level, streamflow, and rating curve data are freely available upon request from the Environment Agency (enquiries@environmentagency.gov.uk). The ERA‐5 data set is freely available at https://confluence.ecmwf.int/display/CKB/ERA5.
